# Genome‐wide association study: Exploring the genetic basis for responsiveness to ketogenic dietary therapies for drug‐resistant epilepsy

**DOI:** 10.1111/epi.14516

**Published:** 2018-07-16

**Authors:** Natasha E. Schoeler, Costin Leu, Simona Balestrini, Jonathan M. Mudge, Charles A. Steward, Adam Frankish, Mary‐Anne Leung, Mark Mackay, Ingrid Scheffer, Ruth Williams, Josemir W. Sander, J. Helen Cross, Sanjay M. Sisodiya

**Affiliations:** ^1^ Department of Clinical and Experimental Epilepsy UCL Institute of Neurology London UK; ^2^ UCL Great Ormond Street Institute of Child Health London UK; ^3^ NIHR University College London Hospitals Biomedical Research Centre UCL Institute of Neurology London UK; ^4^ Chalfont Centre for Epilepsy Chalfont St Peter UK; ^5^ European Molecular Biology Laboratory Wellcome Genome Campus European Bioinformatics Institute Cambridge UK; ^6^ Wellcome Genome Campus Congenica Ltd Cambridge UK; ^7^ Children's Neurosciences Centre Guy's and St Thomas’ NHS Foundation Trust London UK; ^8^ Department of Paediatrics The University of Melbourne Royal Children's Hospital Melbourne Vic. Australia; ^9^ Murdoch Children's Research Institute Melbourne Vic. Australia; ^10^ Epilepsy Research Centre Department of Medicine The University of Melbourne Austin Health Melbourne Vic. Australia; ^11^ Austin Health Florey Institute of Neurosciences and Mental Health Melbourne Vic. Australia; ^12^ Stichting Epilepsie Instellingen Nederland (SEIN) Heemstede The Netherlands; ^13^ Great Ormond Street Hospital for Children London UK; ^14^ Young Epilepsy Lingfield UK

**Keywords:** biomarker, *CDYL*, genetics, high‐fat, low‐carbohydrate

## Abstract

**Objective:**

With the exception of specific metabolic disorders, predictors of response to ketogenic dietary therapies (KDTs) are unknown. We aimed to determine whether common variation across the genome influences the response to KDT for epilepsy.

**Methods:**

We genotyped individuals who were negative for glucose transporter type 1 deficiency syndrome or other metabolic disorders, who received KDT for epilepsy. Genotyping was performed with the Infinium HumanOmniExpressExome Beadchip. Hospital records were used to obtain demographic and clinical data. KDT response (≥50% seizure reduction) at 3‐month follow‐up was used to dissect out nonresponders and responders. We then performed a genome‐wide association study (GWAS) in nonresponders vs responders, using a linear mixed model and correcting for population stratification. Variants with minor allele frequency <0.05 and those that did not pass quality control filtering were excluded.

**Results:**

After quality control filtering, the GWAS of 112 nonresponders vs 123 responders revealed an association locus at 6p25.1, 61 kb upstream of *CDYL* (rs12204701, *P =* 3.83 × 10^−8^, odds ratio [A] = 13.5, 95% confidence interval [CI] 4.07‐44.8). Although analysis of regional linkage disequilibrium around rs12204701 did not strengthen the likelihood of *CDYL* being the candidate gene, additional bioinformatic analyses suggest it is the most likely candidate.

**Significance:**

*CDYL* deficiency has been shown to disrupt neuronal migration and to influence susceptibility to epilepsy in mice. Further exploration with a larger replication cohort is warranted to clarify whether *CDYL* is the causal gene underlying the association signal.

**Key Points**

- The minor allele of rs12204701 is associated with a poor response to KDT
- *CDYL*, 61kb upstream from the association signal, has been implicated in seizure‐related neurodevelopmental disorders
- Replication analyses with a larger cohort are needed

## 1 INTRODUCTION

Ketogenic dietary therapies (KDTs), including the classical, medium chain triglyceride (MCT), and modified ketogenic diets and the low glycemic index treatment (LGIT), are a group of high‐fat, low‐carbohydrate diets that have been used effectively as treatment options for people with drug‐resistant epilepsy since the early 1900s.^1^, ^2^ Excepting specific metabolic disorders (glucose transporter type 1 [GLUT1] deficiency syndrome and pyruvate dehydrogenase complex deficiency), no accurate predictors of response to KDTs are known.^3^ KDTs are resource‐intensive, require dietary restriction, and can cause adverse side effects. The ability to predict response to KDTs would allow targeting of limited dietetic and other medical resources, prioritizing those who are more likely to respond, thus also promoting dietary treatment earlier in the course of epilepsy.

Certain epilepsies, such as epilepsy with myoclonic‐atonic seizures, tuberous sclerosis complex, and Dravet syndrome,^4^ generally respond well to KDT. Tuberous sclerosis complex and Dravet syndrome are caused by single gene mutations. “Highly refractory genetic epilepsies” have an excellent response to KDT.^5^

KDTs cause gene expression changes in animal models in the brain, liver, and white adipose tissue.^6^, ^7^, ^8^, ^9^ A genetic basis for differential response to KDT has been shown in patients with Alzheimer's disease: daily administration of the ketogenic agent AC‐1202 for 90 days resulted in significant differences in serum β‐hydroxybutyrate levels and Alzheimer's disease Assessment Scale (ADAS) – Cognitive subscale scores compared to placebo, most notably in people not carrying the apolipoprotein E4 (*APOE4*) allele.^10^ Consumption of an MCT drink, compared to placebo, has led to improved cognitive performance in *APOE4*‐ but not *APOE4*+ subjects with Alzheimer's disease.^11^ Strain‐specific responsive to KDTs in terms of seizure threshold was shown in an animal study.^12^

Individual genetic variation may thus influence the efficacy of KDT on seizure control. We showed that common variants in *KCNJ11* and *BAD* do not influence KDT response.^13^ Here, we conducted a genome‐wide association study (GWAS) to screen for other common variants that may influence KDT response and to identify biologic pathways not previously associated with KDT.

## 2 METHODS

### 2.1 Ethics and recruitment

The project had relevant ethics committees or institutional review board approval. Informed consent was obtained from all study participants or their parents.

Participants were recruited from April 2011 to December 2012 from the following sites: Great Ormond Street Hospital for Children, London; National Hospital for Neurology and Neurosurgery, London; Evelina Children's Hospital, London; St George's Hospital, London; Young Epilepsy (including Matthew's Friends clinics for Ketogenic Dietary Therapies), Surrey; Birmingham Children's Hospital, Birmingham; Addenbrooke's Hospital, Cambridge; Alder Hey Children's Hospital, Liverpool; Bristol Royal Hospital for Sick Children, Bristol, all in the UK; Austin Health and The Royal Children's Hospital, Melbourne, Australia.

Study inclusion criteria were as follows: individuals aged ≥3 months who were either following KDT, who were soon to be commencing KDT, or who had followed KDT in the past for their epilepsy. Exclusion criteria were as follows: individuals who discontinued KDT before the 3‐month point due to lack of tolerability (but those who discontinued KDT before the 3‐month point due to lack of response or seizure increase were not excluded); individuals with known GLUT1 deficiency, pyruvate dehydrogenase complex deficiency, or other metabolic disorders; and individuals with progressive myoclonic epilepsies (as lack of response may be due to the progressive nature of the condition).

In the UK clinics and Austin Health, every individual eligible for recruitment was invited to participate. All cases from The Royal Children's Hospital, Australia, were recruited retrospectively.

### 2.2 Categorization of KDT response

KDT response was defined as a function of seizure frequency, as published previously.^13^, ^14^ Response was estimated in 28‐day epochs prior to starting the diet (baseline) and prior to 3‐month follow‐up after the start of KDT. Clinic letters and seizure diaries, where already used as part of clinical monitoring, were used to estimate seizure frequency at each time point. The calculation used to determine percentage reduction in seizure frequency was as follows: [(*a*‐*b*)/*a*]*100, where *a* = number of seizures in the 28 days prior to KDT initiation; *b* = number of seizures in the 28 days preceding the 3‐month point.

Cases with ≥50% seizure reduction were classified as “responders”; those with <50% seizure reduction were “nonresponders.” A ≥50% seizure reduction was viewed as clinically useful in this drug‐resistant cohort and has been used as a measure of response to KDT in previous studies.^1^, ^2^ Response at 3‐month follow‐up was used as the primary phenotypic endpoint. There was no minimum time period for which participants should have continued KDT, to enable inclusion of extreme nonresponders who may have discontinued dietary treatment within days/week.

### 2.3 Effect of demographic, clinical, and biochemical factors on KDT response

Clinical and demographic data were obtained from hospital records. For individuals with these data available, the effect of clinical/demographic factors on KDT response at 3‐month follow‐up was assessed by *t*‐test (for continuous variables all were normally distributed) or Pearson χ^2^ (for categorical or binary variables), as appropriate. No test was performed for epilepsy syndrome, as the numbers within each group were small (the majority of the cohort had no syndromic diagnosis). The association between selected biochemical parameters taken at baseline, at 3‐month follow‐up, and the difference in results at these two time points, with KDT response at 3 months, was also assessed by *t*‐test or Pearson χ^2^, as appropriate. Biochemical parameters were selected based on their role in fat and carbohydrate metabolism, as described previously.^15^ A Bonferroni‐corrected significance threshold was calculated, based on an alpha of 0.05 and the number of tests conducted. Univariate logistic regression analysis was performed, considering KDT response at 3‐month follow‐up as outcome variable and each clinical, demographic, and biochemical factor was tested as an independent variable. Associations with *P* < .05 were used to build a multivariate model. Variables with high collinearity (variance inflation factor >5) were excluded from the multivariate model. We estimated odds ratios (ORs) and 95% confidence intervals (CIs). Data analysis was performed using the Stata/IC 11.1 Statistical package (StataCorp, College Station, TX, USA).

### 2.4 Genotypic data collection

DNA was extracted from blood drawn at the same time as routine clinical monitoring. *SLC2A1* was sequenced in all samples to formally exclude the possibility of GLUT1 deficiency syndrome. Samples were genotyped with the Infinium HumanOmniExpressExome Beadchip (Illumina Inc, San Diego, CA, USA). See Data S1 for details.

### 2.5 Genome‐wide association study

Quality control (QC) filtering was applied at individual‐ and variant‐level using PLINK (v1.90,^16^ https://www.cog-genomics.org/plink/1.9/), KING: Kinship‐based INference for Gwas (http://people.virginia.edu/~wc9c/KING/ 17), and GenomeStudio (v2011.1, Illumina Inc). We removed individuals according to 4 quality control (QC) criteria: (1) discordant sex information; (2) overall single nucleotide polymorphisms (SNPs) missingness rate >2%; (3) low (<25%) or high (>33%) heterozygosity rate of autosomal SNPs; and (4) duplicated or related individuals exceeding a proportion of alleles shared identically by descent according to third‐degree relatives and higher (kinship coefficient >0.0442).

SNPs were excluded according to 4 QC criteria: (1) cluster separation <0.3 and Het‐excess values between −0.1 and −1 and between 0.1 and 1 after manual reclustering of SNPs with >1% “no calls” in GenomeStudio; (2) minor allele frequency (MAF) <1% in cases and controls; (3) per‐SNP missingness rate >2% in cases or controls; and (4) deviation from the Hardy‐Weinberg equilibrium (HWE) with *P* < 1 x 10^−20^ in cases and *P* < 1 x 10^−5^ in controls. The QC filtered dataset was aligned to the 1000 Genomes (1000G) dataset using the tool GenotypeHarmonizer (v1.4.20^18^), to exclude strand coding issues during the step of imputation.

Further details regarding quality control filtering are given in Data S1.

Imputation of the QC‐filtered genotype data was performed using Minimac3 with the reference panel of the Haplotype Reference Consortium (HRC r1.1 2016^19^), as implemented on the Michigan Imputation Server.^20^ Phasing was performed for the autosomes using Eagle (v2.3^21^) and ShapeIT (v2.r790^22^) for the X chromosome. The Minimac3 output in variant call format dosage format was converted to PLINK dosage format using DosageConvertor v1.0.4 (https://genome.sph.umich.edu/wiki/DosageConvertor) and converted to hard calls using a threshold of 0.9 in PLINK. Finally, the imputed dataset was QC filtered using following exclusion criteria: (1) imputation accuracy: Rsq≤0.3; (2) estimated *R*‐squared in leave‐one‐out analysis: LooRsq≤0.3; (3) call rate (CR) ≤0.95 after applying a hard call threshold = 0.9; and (4) MAF≤0.01.

Power calculations were performed using PGA Power Calculator.^23^ A codominant model was used, assuming 80% power and a disease prevalence equivalent to KDT response rate. The estimated prevalence of treatment‐resistant epilepsy (∼35% of 0.5% of the population), the usual subject population considered for KDT, was used in place of “disease prevalence.”

GWAS of the KDT response at 3‐month follow‐up as the phenotype was conducted within all samples using a linear mixed model, as implemented in FaST‐LMM (v2.07^24^). The linear mixed model captures all sources of structure (cryptic relatedness and population stratification) based on estimates of the genetic relatedness of individuals. The relationship matrix was calculated by FaST‐LMM based on a subset of SNPs, filtered using following SNP exclusion criteria: (1) CR>0.98; (2) MAF>0.01; (3) SNPs in regions known for high linkage disequilibrium (LD) (Table S1); and (4) SNPs with LD *R* ^2^ > 0.2 within a window of 20 Mb.

### 2.6 Manual investigation of variation

Two aspects for potential functionality of detected variation were investigated. First, the region containing variants of interest was manually reannotated to ensure that no gene features had been missed.^25^ Second, transcriptomics data were employed to investigate potential functionality of associated variants (see Data S1 for details).

## 3 RESULTS

### 3.1 SLC2A1 sequencing

*SLC2A1* sequencing failed in 8 individuals due to low quantity or quality DNA. As published previously,^14^ one individual had a putatively deleterious variant in *SLC2A1*; this individual was subsequently diagnosed with GLUT1 deficiency syndrome and was not included in the GWAS. Two further individuals (one extreme nonresponder who discontinued KDT immediately and one with a variable response to KDT, who remained on KDT long‐term) harbored a missense variant in *SLC2A1*, but these were both predicted to be tolerated by functional prediction algorithms. These 2 individuals were included in the GWAS. One of these variants (c.10A>G) was not found in ExAC, 1000G, GnomAD, or ESP6500; the other variant (c.1408G>C) was found in ExAC (allele frequency 0.00006591), 1000G (allele frequency 0.001), and GnomAD (allele frequency 4.068 x 10^−6^) but not in ESP6500. All synonymous and noncoding variants with MAF <2% were analyzed with Alamut (Interactive Biosoftware, LLC, Rouen, France), but none were predicted to affect splicing (removal of intronic regions located between exons for production of RNA).

### 3.2 Cohort demographics

The cohort consisted of 252 individuals with diet response data, excluding the individual diagnosed with GLUT1 deficiency syndrome. Demographic and clinical data are given in Table 1. Before quality control filtering, the cohort consisted of 122 nonresponders and 130 responders at the 3‐month point. Two hundred six (82%) of this cohort were Caucasian (self‐reported).

**Table 1:** Clinical and demographic characteristics of cohort (for cases with diet response data, n = 252)

Gender	Male, n = 131 (52%) Female, n = 121 (48%)
Ethnicity	Caucasian, n = 206 (82%)
	African, n = 4 (1.6%)
	Middle Eastern, n = 4 (1.6%)
	Central/South Asian, n = 13 (5%)
	East Asian, n = 2 (0.8%)
	Black and Caucasian mix, n = 18 (7%)
	East Asian and Caucasian mix, n = 3 (1.2%)
	South Asian and Caucasian mix, n = 2 (0.8%)
Age at seizure onset (years) median (IQR)	0.67 (0.2‐2) (unknown for 1 case)
Age at diet onset (years) median (IQR)	5.70 (3.2‐9.9)
Cause of epilepsy^a^	Genetic, n = 31 (12%)
	Structural‐metabolic, n = 71 (28%)
	Unknown cause, n = 150 (60%)
Epilepsy syndrome^a^	Dravet syndrome/severe myoclonic epilepsy of infancy, n = 15 (6%);
	Lennox‐Gastaut syndrome/LGS‐spectrum, n = 13 (5.2%)
	Childhood absence epilepsy, n = 3 (1.2%)
	Juvenile myoclonic epilepsy, n = 2 (0.8%)
	Juvenile absence epilepsy, n = 3 (1.2%)
	Epilepsy with myoclonic‐atonic seizures (Doose syndrome), n = 14 (5.6%)
	Epilepsy with myoclonic absences, n = 1 (0.4%)
	Epilepsy with myoclonic‐atonic seizures and myoclonic absences, n = 2 (0.8%)
	Myoclonic epilepsy (unspecified), n = 7 (2.8%)
	Epilepsy of infancy with migrating focal seizures, n = 3 (1.2%)
	Ohtahara syndrome, n = 1 (0.4%)
	West syndrome, n = 16 (6.3%)
	Undiagnosed, n = 172 (68.2%)
Number of AEDs at diet onset mean [95% CI]	2.34 [2.22—2.46] (unknown for 1 case)
Number of failed AEDs prior to diet onset mean [95% CI]	6.61 [6.28‐6.94] (unknown for 3 cases)
Diet type (at 3‐month point)^b^	Classical ketogenic diet, n = 165 (65.5%)
	Medium chain triglyceride ketogenic diet, n = 48 (19%)
	Modified ketogenic diet, n = 38 (15.1%)
	Unknown, n = 1 (0.4%)
Feed	Oral, n = 171 (67.9%)
	Tube, n = 64 (25.4%)
	Oral and tube, n = 16 (6.3%)
	Unknown, n = 1 (0.4%)

IQR, interquartile range.

a Cause of epilepsy (genetic, structural/metabolic, unknown) and epilepsy syndromes have been classified according to Berg et al, 2010.^36^

b No patients were following the low glycemic index treatment, as this was not offered as an option at the study sites. If a patient transitioned to a different diet type before the 3‐month point, the new/second diet type was considered this individual's diet type.

### 3.3 Effect of demographic, clinical, and biochemical factors on KDT response

No clinical, demographic, or biochemical factor was found to affect KDT response at 3‐month follow‐up after correction for multiple testing (the significance threshold was set at 0.002, based on an alpha of 0.05 and 23 tests [9 clinical/demographic factors and 14 biochemical parameters]), as shown in Tables S2‐S5. The lowest *P* value was for acetylcarnitine at baseline (16.45 μmol/L in the responders vs 12.38 μmol/L in nonresponders, *P* = .0034, *t*‐test). This is a *P* value similar to that reported in our previously published work on the same cohort, which showed a significant association between KDT response at 3‐month follow‐up and baseline acetylcarnitine^15^; a greater number of tests have been used in present analyses, which may explain why the *P* value did not reach significance after correcting for multiple testing.

Univariate logistic regression analysis showed significant association between KDT response at 3‐month follow‐up and: number of failed AEDs (OR 0.89, 95% CI 0.81‐0.99, *P* = .026), free carnitine (OR 1.03, 95% CI 1.00‐1.06, *P* = .040), acetylcarnitine (OR 1.09, 95% CI 1.03‐1.16, *P* = .006), propionyl carnitine (OR 2.15, 95% CI 1.06‐4.36, *P* = .033), and palmitoylcarnitine (OR 6.25, 95% CI 1.17‐33.54, *P* = .033) at baseline, and free carnitine (OR 1.03, 95% CI 1.00‐1.06, *P* = .028), acetylcarnitine (OR 1.04, 95% CI 1.00‐1.09, *P* = .033), and palmitoylcarnitine (OR 3.38, 95% CI 1.01‐11.33, *P* = .049) at 3‐month follow‐up. Multivariate logistic regression showed only a significant association for KDT response with palmitoylcarnitine at 3‐month follow‐up (OR 4.59, 95% CI 1.06‐19.92, *P* = .041). This parameter was not included as a covariate in the GWAS, as it did not reach statistical significance after correction for multiple testing in association tests.

### 3.4 Genome‐wide association study

Three subjects were not genotyped with the Infinium HumanOmniExpressExome Beadchip, due to a delay in receiving DNA samples. Fourteen subjects were removed from the association analysis due to relatedness to another study participant. Three subjects were removed due to excess/reduced heterozygosity rates.

Following quality control filtering, 4,819,069 SNPs, 112 nonresponders and 123 responders were included in the single‐variant GWAS.

**Figure 1:**
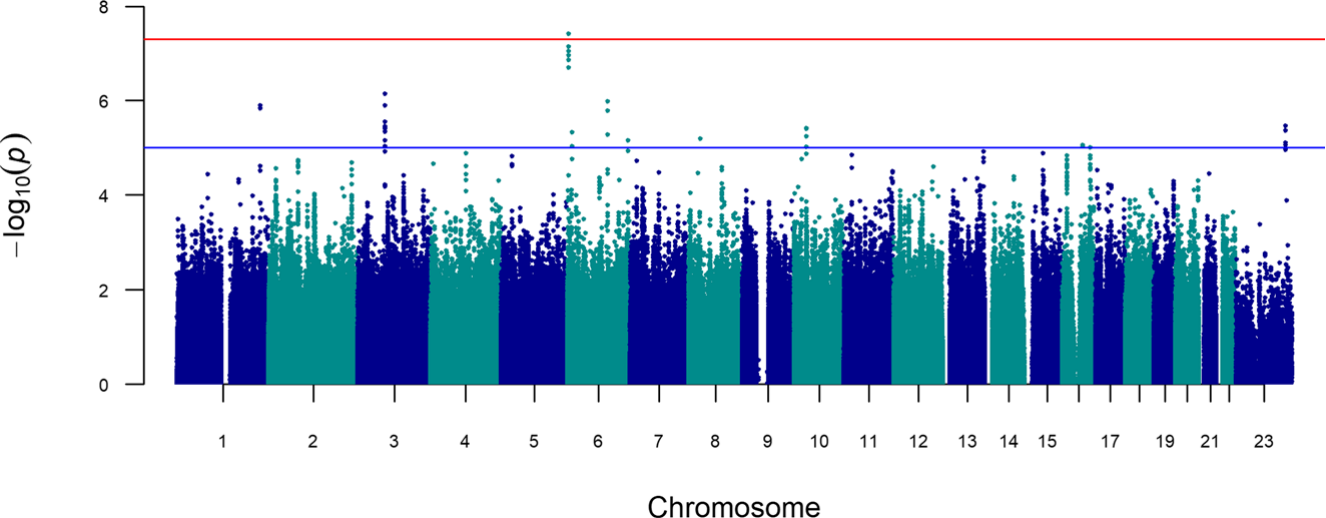
Manhattan plot of genome‐wide association results. X‐axis represents genomic location; y‐axis represents −log10 of unadjusted *P* values for each single nucleotide polymorphisms (SNP). Red line, genome‐wide significance level of 5 x 10^−8^. Blue line, suggestive significance level of 1 x 10^−5^

Using a linear mixed model, which provides robust correction for familial or cryptic relatedness and population stratification, association emerged at 6p25.1, 61 kb upstream of *CDYL* (rs12204701, unadjusted *P* = 3.83 x 10^‐8^, OR [A] = 13.5, 95%‐CI 4.07‐44.8). The minor allele, A, was more frequent in nonresponders than responders. According to 1000 Genomes, the MAF (A) of rs12204701 is 0.0938/470. Figure 1 shows the Manhattan plot of the association results in genomic context.

**Figure 2:**
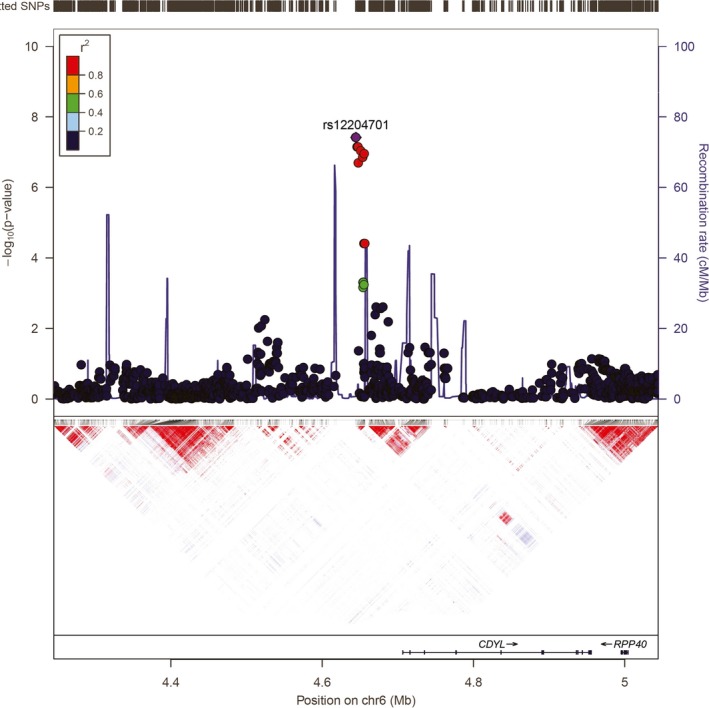
Regional association plot and linkage disequilibrium (LD) map for rs12204701 ± 500 bp. In the association plot, the left y‐axis represents −log10 (*P* values) for association with 3‐month ketogenic dietary therapy (KDT) response; the right y‐axis represents the recombination rate; the x‐axis represents base‐pair positions along the chromosome (human genome build 37). The top variant, rs12204701, is shown in purple; the rest of the variants are colored according to their LD *r* ^2^ value with rs12204701. In the LD map, LD is indicated as D’/LOD, ranging from red to blue according to the strength of evidence of LD. LD pattern is based on genotype data obtained from this study. Confidence interval minima for strong LD: lower: 0.7, upper 0.98; upper confidence interval maximum for strong recombination: 0.9; fraction of strong LD in informative comparisons are at least 0.95; markers with <0.05 minor allele frequency (MAF) are excluded

Investigation of the regional linkage disequilibrium (LD) structure of the associated region revealed that the top hit, rs12204701, is in an LD block next to, but not encompassing the gene Chromodomain Y‐like (*CDYL*) and separated by several recombination hot spots (Figure 2).

**Figure 3:**
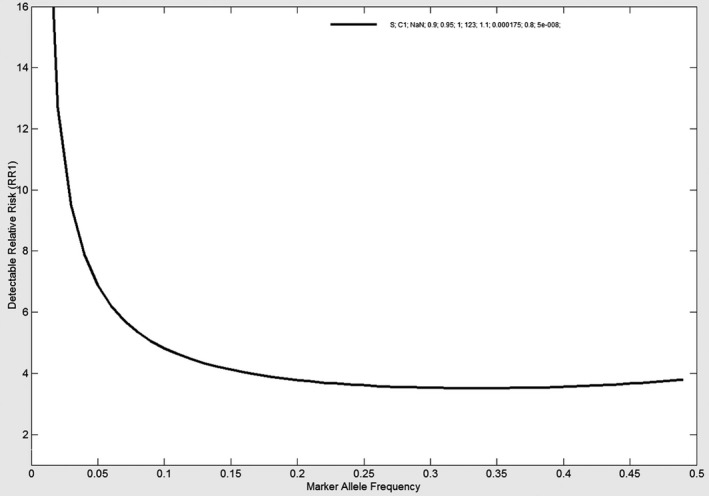
Detectable relative risk and disease allele frequency curves for 3‐month ketogenic dietary therapy (KDT) response cohort, with 80% power, assuming *r* ^2^ of 0.9 between genotyped marker and causal variant, a disease prevalence of 0.00175, alpha = 5 x 10^−8^, 112 cases and control‐to‐case ratio of 1.10

Figure 3 shows the detectable relative risk of variants with varying MAF in the GWAS cohort, using a codominant model, with 80% power. Variants with a MAF of approximately ≥0.1 and a relative risk of 5 could be detected with our sample size. A larger cohort would be needed to detect variants with smaller relative risks or lower allele frequencies.

**Figure 4:**
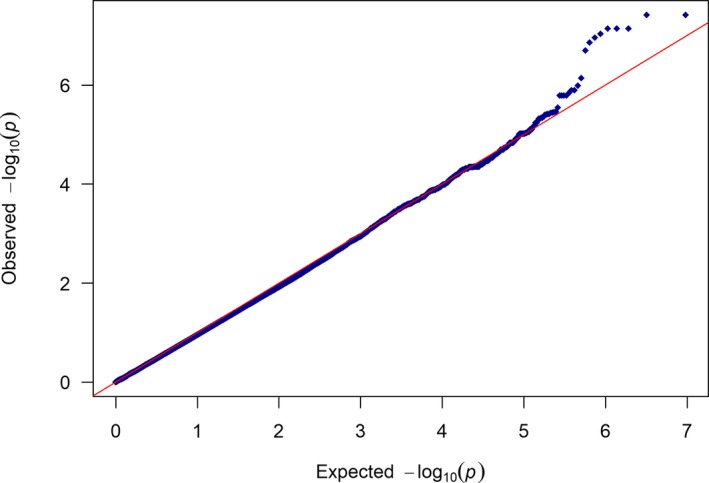
Quantile‐quantile plot of genome‐wide association study (GWAS) results from Fisher's exact test

Genomic control λ was 0.9 and the quantile‐quantile plot (Figure 4) indicated deviation from the null hypothesis of no association only in the upper tails, corresponding to the SNPs with strongest evidence for association. This suggests the absence of confounding factors.

### 3.5 Manual investigation of variant

We did not find any strong evidence to support the transcription of this variant, either as part of *CDYL* or as an independent gene, although Intropolis data suggest the presence of a long noncoding RNA on the negative strand. Epigenetic, open chromatin, and transcription factor‐binding data indicate that the variant is located on the 5′ edge of a putative enhancer region in certain cell types, with consistent DNAseq hypersensitivity across a range of experiments, and rich transcription factor‐binding data. Although these annotations do not overlap with the variant, they are found within the same LD block, as indicated by the red‐shaded triangle downstream of rs12204701 in the LD map in Figure 2. We find that the variant is consistently located with the same topologically associated domain (TAD) as *CDYL* across a wide range of experiments in different cell types, and that *CDYL* is the only protein‐coding gene found within this domain.

## 4 DISCUSSION

We conducted a GWAS for responsiveness to KDT. Service provision for KDT is limited, even in resource‐rich countries, and so the numbers of cases available for inclusion, with adequate data and a limited collection timeframe, is inevitably small. Despite this, our study is reasonably powered to identify common variation of large effect size, which is the most important for clinical prediction and mechanistic understanding. We show that the minor allele of rs12204701 is associated (*P =* 3.83x10^‐8^, odds ratio [A] = 13.5) with poor response (<50% seizure reduction) to KDT at 3‐month follow‐up. Our GWAS consisted mainly (but not exclusively) of participants of European ancestry and so our results may not be applicable to other populations.

rs12204701 is a noncoding SNP located 61 kb upstream of *CDYL*, and so may have a regulatory function. *CDYL* is a transcriptional corepressor that is expressed ubiquitously in humans and that is required for the transmission/restoration of repressive histone marks, which is critical for the maintenance of cell identity.^26^ *CDYL* drives neuronal migration^27^ and regulates activity‐dependent intrinsic neuronal plasticity.^28^ It transcriptionally represses *SCN8A*, the gene encoding Nav1.6 sodium channels, causing a reduction in axonal Nav1.6 currents, the dysfunction of which are associated with epilepsy, including severe developmental and epileptic encephalopathies, and other neurologic and psychiatric brain disorders.^28^ *CDYL* regulates dendrite morphogenesis in rat/mouse hippocampal neurons^29^ and its deficiency increases excitability of cortical pyramidal neurons and susceptibility to epilepsy in mice.^27^ Of the 22 proteins found to interact with CDYL, most play a role in transcriptional repression.^30^ *CDYL* is involved in the repression of transcription of the proto‐oncogene *TrkC*, which is important for suppression of cellular transformation^30^; this is of interest because of the neuroprotective properties of KDT and the potential role of apoptosis in its mechanisms of action in this regard.^31^, ^32^ SNPs located in the region encompassing the association signal, between *KU‐MEL‐3* and *CDYL,* have also been associated with phenotypic traits relevant to metabolism of high‐fat, low‐carbohydrate diet: cholesterol levels^33^ and susceptibility to type 2 diabetes.^34^ rs12204701 may tag other SNPs or even copy number variants that may influence KDT response. Based on an assumption that promoter‐enhancer interactions can occur only within specific TADs,^35^ *CDYL* would appear to be the most likely target for this putative regulatory region. Our leading hypothesis is therefore that the variant may affect an enhancer element that regulates *CDYL* or is in linkage disequilibrium with a variant affecting an enhancer of *CDYL*.

In conclusion, our analyses in patients who are negative for GLUT1 deficiency syndrome (caused by *SLC2A1* mutation) indicate that rs12204701 is associated with poor response to KDT. *CDYL* is, due to its vicinity and function, the most likely candidate gene. The putative effect of genetic variation on KDT response remains largely unknown, other than in specific metabolic disorders. We recognize that our study is of small numbers of participants, but nevertheless has demonstrated an association we consider important to bring to a wider audience. The relevance of rs12204701 merits further exploration with a replication cohort, ideally with a large enough cohort size to allow sufficient power to detect effects from less common variants or those with lesser effect sizes, and perhaps also to permit appropriately powered sub‐analyses of people with distinct epilepsy etiologies and syndromes.

## DISCLOSURE OF CONFLICT OF INTEREST

NS is funded by Vitaflo for her current post. M‐AL has meeting support from Nutricia Metabolics and BioMarin Pharmaceuticals Inc, and honoraria to the department from BioMarin Pharmaceuticals Inc. The Evelina London Children's Hospital Dietary Epilepsy Service has been supported financially in the past by The Daisy Garland. IES has received honoraria from UCB, Eisai, GlaxoSmithKline (GSK), Nutricia, and Biomarin. She has cowritten “Ketocooking” with JHC; funds are donated to the KDT program. JWS has received research grants and honoraria from UCB, Eisai, and GSK, which are involved in the manufacturing of antiepileptic drugs. JHC has received funds to the department for research into the ketogenic diet from Vitaflo. Honoraria for speaking have been donated to the department from Nutricia, Eisai, UCB, Zogenix, and GW Pharma. JHC has cowritten a cookery book, “Ketocooking,” funds from the sale of which are donated to the department. SMS has received meeting support or honoraria Vitaflo and Nutricia. The remaining authors have no conflict of interest in relation to this work. We confirm that we have read the Journal's position on issues involved in ethical publication and affirm that this report is consistent with those guidelines.
