# Supplementary material for: Genome‐wide association study: Exploring the genetic basis for responsiveness to ketogenic dietary therapies for drug‐resistant epilepsy
Source: Epilepsia. 2018 Jul 16;59(8):1557–66. doi: 10.1111/epi.14516 (PMC6099477; doi:10.1111/epi.14516)
Supplement: Supplementary file 1 [file EPI-59-1557-s001.docx]

**Supplementary Methods**

*Genotypic data collection*

Samples taken in the UK had DNA extracted either by Autogen (AutoGen Inc, Hollister, Massachusetts, USA) for samples 1.5ml-4ml, or by Fujifilm (FUJIFILM corporation, Tokyo, Japan) for samples 0.5ml-2.5ml. For blood samples taken at the Royal Children's Hospital, Melbourne, DNA was extracted locally using Maxi Qiagen kits.

For *SLC2A1* sequencing, DNA was quantified using a Qubit**®** Fluorometer (Life Technologies Corporation). 250ng of DNA was used in the Illumina TruSeq Custom Amplicon Library Preparation protocol, according to manufacturer’s instructions (TruSeq Custom Amplicon Library Preparation Guide, © 2013 Illumina, Inc).

Samples were genotyped with the Infinium HumanOmniExpressExome Beadchip (Illumina Inc, San Diego, USA) in two batches at AROS Applied Biotechnology A/S, Denmark. Genotype calling was performed using Illumina GenomeStudio software (v2011.1, Illumina Inc, San Diego, USA) with the Genotyping module (v1.0, Illumina Inc, San Diego, USA), according to manufacturer’s instructions (Technical Note: DNA Analysis, Infinium® Genotyping Data Analysis, www.illumina.com). Genotyping data were imported into PLINK (v1.90, https://www.cog-genomics.org/plink/1.9/, 07, <http://zzz.bwh.harvard.edu/plink/>,^1^) for quality control filtering.

**GWAS**

*Per-individual quality control exclusion criteria:*

The sex of participants was imputed from PLINK. Homozygosity estimates are calculated across all X-chromosome SNPs for each individual; a male call is made when homozygosity estimate>0.8 and a female call is made when homozygosity estimate<0.2. Cases with a mismatch between sex reported in the phenotype file and imputed sex were to be removed.

Individuals with >2% missing SNP data were removed.

The observed number of homozygous genotypes and total number of non-missing genotypes were calculated in PLINK*.* The proportion of heterozygous SNPs for each individual, stratified by self-reported ethnicity, was plotted in *Microsoft Excel 2010* (v. 14, Microsoft, Washington, USA), as shown in Supplementary Figure 1. Visual inspection of this plot enabled identification and elimination of outliers in each ethnic group.

Supplementary Figure 1: Mean heterozygosity rates per individual, separated by ethnicity

**Ethnicity Key**

1 – Caucasian

2 – African

3 - Middle Eastern

4 - Central/South Asian

5 - East Asian

6 - Black and Caucasian mix

7 - East Asian and Caucasian mix

8 - South Asian and Caucasian mix

Duplicate and related individuals were identified using KING: Kinship-based INference for Gwas (<http://people.virginia.edu/~wc9c/KING/>, ^2^). Individuals exceeding a proportion of alleles shared identically by descent, equivalent to third-degree relatives and higher (kinship co-efficient >0.0442) were removed from analyses. The subset of SNPs used by FaST-LMM (v2.07^3^) to calculate the relationship matrix was filtered according to the following exclusion criteria: i) CR>0.98; ii) MAF>0.01; iii) SNPs in regions known for high linkage disequilibrium (LD) (Supplementary Table 1); iv) SNPs with LD R^2^ > 0.2 within a window of 20Mb.

**Supplementary Table 1.** Known high-LD regions excluded from the relationship matrix.

| **Chromosome** | **Start position (NCBI build 36)** | **End position (NCBI build 36)** |
| --- | --- | --- |
| 1 | 48060567 | 52060567 |
| 2 | 85941853 | 100407914 |
| 2 | 134382738 | 137882738 |
| 2 | 182882739 | 189882739 |
| 3 | 47500000 | 50000000 |
| 3 | 83500000 | 87000000 |
| 3 | 89000000 | 97500000 |
| 5 | 44500000 | 50500000 |
| 5 | 98000000 | 100500000 |
| 5 | 129000000 | 132000000 |
| 5 | 135500000 | 138500000 |
| 6 | 25500000 | 33500000 |
| 6 | 57000000 | 64000000 |
| 6 | 140000000 | 142500000 |
| 7 | 55193285 | 66193285 |
| 8 | 8000000 | 12000000 |
| 8 | 43000000 | 50000000 |
| 8 | 112000000 | 115000000 |
| 10 | 37000000 | 43000000 |
| 11 | 46000000 | 57000000 |
| 11 | 87500000 | 90500000 |
| 12 | 33000000 | 40000000 |
| 12 | 109521663 | 112021663 |
| 20 | 32000000 | 34500000 |

The cluster plots of the top associated SNPs were manually inspected using GenomeStudio to ensure accurate genotyping.

*Manual investigation of variation*

Potential missing annotation could include unannotated alternatively spliced (AS) transcripts that extend an adjacent gene, connecting it with a variant, either in an exonic region of novel CDS or 5’ UTR or proximal regulatory region. Similarly, other unannotated genic features, such as long non-coding RNAs and small RNAs, were also checked. As such, variants were analysed in the genomic context using a broad range of experimentally-derived datasets.

Secondly, for transcriptomics, traditional evidence sources (i.e. cDNA and EST libraries) were queried, alongside short-read sequencing datasets including those from ENCODE^4^, the Human Proteome Atlas^5^, Intropolis RNAseq-supported introns^6^ and the cap analysis of gene expression (CAGE) libraries from FANTOM5^7^. Pseudo-long read (SLRseq) datasets^8^, which are brain-derived, and novel PacBio datasets produced from a variety of normal tissues including brain (<http://www.biorxiv.org/content/early/2017/06/16/105064)> were also utilised. The relative position of variants relative to distal regulatory regions and any contacts to the promoters of nearby protein-coding genes were also assessed. The local context of the variant was investigated based on epigenetics data, employing the resources of the Ensembl Regulatory Build^9^, ENCODE ChIP-seq resources^4^. We also examined the 3D structure of the genomic region using the Hi-C datasets made available through the Penn State 3D Genome Browser (<http://www.biorxiv.org/content/early/2017/02/27/112268>).

Supplementary Table 2: Relationship between KDT response at 3-month follow-up and clinical and demographic factors

| Factor | Number of responders (≥50% seizure reduction) with data available | Number of non-responders (<50% seizure reduction) with data available | P value |
| --- | --- | --- | --- |
| Gender | 130 | 122 | 0.515 |
| Ethnicity | 130 | 122 | 0.619 |
| Age of seizure onset (years) | 130 | 121 | 0.870 |
| Age of diet onset (years) | 130 | 122 | 0.198 |
| Cause of epilepsy | 130 | 122 | 0.649 |
| Number of AEDs taken at diet onset | 129 | 122 | 0.668 |
| Number of failed AEDs prior to diet onset | 128 | 121 | 0.024 |
| Diet type | 130 | 121 | 0.157 |
| Feed | 129 | 122 | 0.408 |

Supplementary Table 3: Relationship between KDT response at 3-month follow-up with biochemical parameters at baseline

|  | Number of responders (≥50% seizure reduction) with data available | Number of non-responders (<50% seizure reduction) with data available | p-value |
| --- | --- | --- | --- |
| Acetoacetate (mmol/L) | 18 | 14 | 0.968 |
| Glucose (mmol/L) | 60 | 44 | 0.424 |
| β-hydroxybutyrate (mmol/L) | 65 | 59 | 0.029 |
| Glucose-ketone index | 41 | 32 | 0.057 |
| Non-esterified fatty acids (mmol/L) | 59 | 54 | 0.515 |
| Free carnitine (μmol/L) | 71 | 66 | 0.037 |
| Acetylcarnitine (μmol/L) | 55 | 53 | 0.003 |
| Propionylcarnitine (μmol/L) | 49 | 36 | 0.027 |
| Butyrylcarnitine (μmol/L) | 49 | 36 | 0.887 |
| Isovalerylcarnitine (μmol/L) | 48 | 36 | 0.592 |
| Hexanoylcarnitine (μmol/L) | 43 | 30 | 0.348 |
| Octanoylcarnitine (μmol/L) | 49 | 37 | 0.102 |
| Tetradecenylcarnitine (μmol/L) | 39 | 27 | 0.802 |
| Palmitoylcarnitine (μmol/L) | 43 | 29 | 0.027 |

Supplementary Table 4: Relationship between KDT response at 3-month follow-up with biochemical parameters at 3-month follow-up

|  | Number of responders (≥50% seizure reduction) with data available | Number of non-responders (<50% seizure reduction) with data available | p-value |
| --- | --- | --- | --- |
| Acetoacetate (mmol/L) | 19 | 10 | 0.510 |
| Glucose (mmol/L) | 49 | 37 | 0.994 |
| β-hydroxybutyrate (mmol/L) | 63 | 57 | 0.168 |
| Glucose-ketone index | 40 | 31 | 0.413 |
| Non-esterified fatty acids (mmol/L) | 59 | 51 | 0.815 |
| Free carnitine (μmol/L) | 58 | 45 | 0.024 |
| Acetylcarnitine (μmol/L) | 49 | 43 | 0.028 |
| Propionylcarnitine (μmol/L) | 43 | 32 | 0.829 |
| Butyrylcarnitine (μmol/L) | 43 | 32 | 0.251 |
| Isovalerylcarnitine (μmol/L) | 43 | 32 | 0.999 |
| Hexanoylcarnitine (μmol/L) | 40 | 28 | 0.862 |
| Octanoylcarnitine (μmol/L) | 43 | 32 | 0.611 |
| Tetradecenylcarnitine (μmol/L) | 34 | 27 | 0.385 |
| Palmitoylcarnitine (μmol/L) | 39 | 28 | 0.040 |

Supplementary Table 5: Relationship between KDT response at 3-month follow-up with the difference in biochemical parameters at baseline and 3-month follow-up

|  | Number of responders (≥50% seizure reduction) with data available | Number of non-responders (<50% seizure reduction) with data available | p-value |
| --- | --- | --- | --- |
| Acetoacetate (mmol/L) | 10 | 8 | 0.403 |
| Glucose (mmol/L) | 32 | 23 | 0.989 |
| β-hydroxybutyrate (mmol/L) | 45 | 37 | 0.024 |
| Non-esterified fatty acids (mmol/L) | 42 | 34 | 0.549 |
| Free carnitine (μmol/L) | 42 | 35 | 0.960 |
| Acetylcarnitine (μmol/L) | 35 | 33 | 0.423 |
| Propionylcarnitine (μmol/L) | 32 | 24 | 0.046 |
| Butyrylcarnitine (μmol/L) | 32 | 24 | 0.064 |
| Isovalerylcarnitine (μmol/L) | 31 | 24 | 0.414 |
| Hexanoylcarnitine (μmol/L) | 29 | 22 | 0.757 |
| Octanoylcarnitine (μmol/L) | 32 | 25 | 0.817 |
| Tetradecenylcarnitine (μmol/L) | 27 | 21 | 0.417 |
| Palmitoylcarnitine (μmol/L) | 29 | 21 | 0.492 |

**Supplementary Table 6: Top hits (P=0.000999) in gene-based GWAS**

| Official Gene Symbol | Gene name | GO Biological Process | GO Molecular Function | Associated KEGG Pathways | | |
| --- | --- | --- | --- | --- | --- | --- |
| *ARRB2* | arrestin beta 2 | negative regulation of protein phosphorylation, G-protein coupled receptor internalization, desensitization of G-protein coupled receptor protein signaling pathway by arrestin, positive regulation of receptor internalization, transcription from RNA polymerase II promoter, signal transduction, transforming growth factor beta receptor signaling pathway, dopamine receptor signaling pathway, brain development, adult walking behavior, positive regulation of gene expression, protein transport, protein ubiquitination, platelet activation, negative regulation of protein ubiquitination, positive regulation of protein ubiquitination, receptor internalization, negative regulation of NF-kappaB transcription factor activity, positive regulation of synaptic transmission, dopaminergic, negative regulation of interleukin-1 beta production, negative regulation of interleukin-12 production, negative regulation of interleukin-6 production, negative regulation of tumor necrosis factor production, positive regulation of peptidyl-serine phosphorylation, negative regulation of toll-like receptor signaling pathway, negative regulation of GTPase activity, negative regulation of smooth muscle cell apoptotic process, follicle-stimulating hormone signaling pathway, negative regulation of cysteine-type endopeptidase activity involved in apoptotic process, proteasome-mediated ubiquitin-dependent protein catabolic process, negative regulation of natural killer cell mediated cytotoxicity, positive regulation of peptidyl-tyrosine phosphorylation, detection of temperature stimulus involved in sensory perception of pain, positive regulation of protein kinase B signaling, negative regulation of protein kinase B signaling, positive regulation of calcium ion transport, Wnt signaling pathway, planar cell polarity pathway, cell chemotaxis, regulation of androgen receptor signaling pathway, positive regulation of ERK1 and ERK2 cascade, negative regulation of release of cytochrome c from mitochondria, chemical synaptic transmission, postsynaptic, positive regulation of DNA biosynthetic process, positive regulation of cardiac muscle cell differentiation | G-protein coupled receptor binding, receptor binding, protein binding, enzyme binding, protein domain specific binding, ubiquitin protein ligase binding, alpha-1A adrenergic receptor binding, alpha-1B adrenergic receptor binding, angiotensin receptor binding, type 1 angiotensin receptor binding, D1 dopamine receptor binding, follicle-stimulating hormone receptor binding, type 2A serotonin receptor binding, platelet activating factor receptor binding, protein complex binding, protein complex scaffold, protein kinase B binding, mitogen-activated protein kinase binding, 14-3-3 protein binding | KEGG pathways: MAPK signaling pathway, Chemokine signaling pathway, Endocytosis, Olfactory transduction, Morphine addiction | | |
| *CCDC7* | coiled-coil domain containing 7 | No GO information available | | | - |  |
| *C19orf35* | chromosome 19 open reading frame 35 | Protein phosphorylation | Protein kinase activity | - | |  |
| *CLTCL1* | clathrin heavy chain like 1 | intracellular protein transport, receptor-mediated endocytosis, mitotic nuclear division, signal transduction, anatomical structure morphogenesis, vesicle-mediated transport, retrograde transport, endosome to Golgi, positive regulation of glucose import | signal transducer activity, structural molecule activity, protein binding | KEGG pathways:  Lysosome, Endocytosis, Synaptic vesicle cycle, Endocrine and other factor-regulated calcium reabsorption, Huntington's disease, Bacterial invasion of epithelial cells | | |
| *FCN1* | Ficolin 1 | complement activation, lectin pathway, cell surface pattern recognition receptor signaling pathway, proteolysis, complement activation, G-protein coupled receptor signaling pathway, protein localization to cell surface, recognition of apoptotic cell, negative regulation of viral entry into host cell, positive regulation of interleukin-8 secretion, |  | - | | |
| *MPO* | myeloperoxidase | response to yeast, hypochlorous acid biosynthetic process, respiratory burst involved in defense response, defense response, response to oxidative stress, aging, response to mechanical stimulus, removal of superoxide radicals, response to food, response to lipopolysaccharide, low-density lipoprotein particle remodeling, defense response to bacterium, hydrogen peroxide catabolic process, negative regulation of apoptotic process, negative regulation of growth of symbiont in host, defense response to fungus, oxidation-reduction process, response to gold nanoparticle | chromatin binding, peroxidase activity, heparin binding, heme binding, metal ion binding | KEGG pathways: Phagosome, Transcriptional misregulation in cancer | | |
| *RAB20* | RAB20, member RAS oncogene family | small GTPase mediated signal transduction, protein transport, phagosome acidification, phagosome-lysosome fusion, regulation of autophagosome assembly | GTP binding | - | | |
| *SLC12A8* | solute carrier family 12 member 8 | potassium ion transport, transmembrane transport | symporter activity | - | | |
| *XPO1* | exportin 1 | ribosomal large subunit export from nucleus, ribosomal small subunit export from nucleus, negative regulation of transcription from RNA polymerase II promoter, protein export from nucleus, intracellular protein transport, sister chromatid cohesion, regulation of centrosome duplication, viral process, protein localization to nucleus, regulation of protein catabolic process, response to drug, regulation of mRNA stability, regulation of protein export from nucleus, mRNA transport, intracellular transport of virus | RNA binding, nuclear export signal receptor activity, transporter activity, nucleocytoplasmic transporter activity, protein binding, Ran GTPase binding, protein domain specific binding | KEGG pathways: Ribosome biogenesis in eukaryotes, RNA transport, Influenza A, HTLV-I infection, Epstein-Barr virus infection | | |

**References**

1 Purcell S, Neale B, Todd-Brown K, et al. PLINK: a tool set for whole-genome association and population-based linkage analyses. Am J Hum Genet 2007; 81: 559-575.

2 Manichaikul A, Mychaleckyj JC, Rich SS, et al. Robust relationship inference in genome-wide association studies. Bioinformatics 2010; 26: 2867-2873.

3 Lippert C, Listgarten J, Liu Y, et al. FaST linear mixed models for genome-wide association studies. Nat Methods 2011; 8: 833-835.

4 Consortium EP. An integrated encyclopedia of DNA elements in the human genome. Nature 2012; 489: 57-74.

5 Fagerberg L, Hallstrom BM, Oksvold P, et al. Analysis of the human tissue-specific expression by genome-wide integration of transcriptomics and antibody-based proteomics. Mol Cell Proteomics 2014; 13: 397-406.

6 Nellore A, Jaffe AE, Fortin JP, et al. Human splicing diversity and the extent of unannotated splice junctions across human RNA-seq samples on the Sequence Read Archive. Genome Biol 2016; 17: 266.

7 Consortium F, the RP, Clst, et al. A promoter-level mammalian expression atlas. Nature 2014; 507: 462-470.

8 Tilgner H, Jahanbani F, Blauwkamp T, et al. Comprehensive transcriptome analysis using synthetic long-read sequencing reveals molecular co-association of distant splicing events. Nat Biotechnol 2015; 33: 736-742.

9 Zerbino DR, Johnson N, Juetteman T, et al. Ensembl regulation resources. Database (Oxford) 2016; 2016: 1-13.
